# Changes in BMI and blood pressure after implementing a complete smoking ban in a medium secure forensic setting: A study from dundrum hospital dublin

**DOI:** 10.1192/j.eurpsy.2021.1021

**Published:** 2021-08-13

**Authors:** A. Roarty, H. Kennedy, M. Davoren

**Affiliations:** 1 Forensic Psyhchiatry, National Forensic Mental Health Service, Central Mental Hospital, Dundrum, Dublin, Ireland; 2 Department Of Psychiatry, Trinity College- Dublin University, Dublin, Ireland

**Keywords:** smoking cessation, medium secure, Blood pressure, BMI

## Abstract

**Introduction:**

In February 2020, the Central Mental Hospital Dundrum moved to a complete ban on cigarette smoking. Concerns were raised that this might represent a ‘restrictive practice’ and that patients might gain weight or see changes in their blood pressure if they were not permitted to smoke.

**Objectives:**

The aim of the study was to ascertain if there were changes in the blood pressure readings or body mass index of a group of patients in a secure forensic hospital after the implementation of a complete campus-wide smoking ban

**Methods:**

All patients (n=20) working with one medium cluster team were included in the study. Demographic details and data pertaining to legal status, diagnosis and length of stay in the hospital were obtained. BMI, blood pressure and medications were reviewed at the time of introduction of the smoking ban, 1^st^ February 2020 and again 5 months later.

**Results:**

All those included in the study were male. The median age was 35 years, most common diagnosis was schizophrenia and mean length of stay was 4.23 years. 20% of patients were prescribed anti-hypertensives at the time of introduction of the smoking ban. All of the patients on anti-hypertensives were overweight. At follow up there was no increase in BMI noted in the patient group. Two patients had dose reductions in anti-hypertensives, three had discontinuation of bronchodilators.

**Conclusions:**

Introducing a campus wide smoking ban in a secure forensic psychiatric hospital is both clinically positive and practically possible. There was no noted increase in incidents in the hospital during this period.

